# Effectiveness of dual antiplatelet de-escalation therapy on the prognosis of patients with ST segment elevation myocardial infarction undergoing percutaneous coronary intervention

**DOI:** 10.1186/s12872-023-03152-8

**Published:** 2023-03-29

**Authors:** Zhigang Zhao, Jingyao Wang, Mengjie Lei, Yachao Li, Yanli Yang, Lei An, Xue Sun, Cairong Li, Zengming Xue

**Affiliations:** Department of Cardiology, Langfang Core Laboratory of Precision Treatment of CAD, Langfang People’s Hospital, Hebei Medical University, Langfang, No. 37, Xinhua Road, 065000 Langfang, China

**Keywords:** Dual antiplatelet, De-escalation, Myocardial infarction, Major adverse cardiovascular and cerebrovascular events, Bleeding

## Abstract

**Aim:**

To investigate the effectiveness of de-escalation of ticagrelor (from ticagrelor 90 mg to clopidogrel 75 mg or ticagrelor 60 mg) on the prognosis of patients with ST segment elevation myocardial infarction (STEMI) undergoing percutaneous coronary intervention (PCI) after 3 months of oral dual antiplatelet therapy (DAPT).

**Methods:**

From March 2017 to August 2021, 1056 patients with STEMI in a single centre, through retrospective investigation and analysis, were divided into intensive (ticagrelor 90 mg), standard (clopidogrel 75 mg after PCI) and de-escalation groups (clopidogrel 75 mg or ticagrelor 60 mg after 3 months of treatment with 90 mg ticagrelor) based on the type and dose of P2Y_12_ inhibitor 3 months after PCI, and the patients had a ≥ 12-month history of oral DAPT. The primary end point was major adverse cardiovascular and cerebrovascular events (MACCEs) during the 12-month follow-up period, including composite end points of cardiac death, myocardial infarction, ischaemia-driven revascularization and stroke. The major safety endpoint was bleeding events.

**Results:**

The results showed that during the follow-up period, there was no statistically significant difference in the incidence of MACCEs between the intensive and de-escalation groups (*P* > 0.05). The incidence of MACCEs in the standard treatment group was higher than that in the intensive treatment group (*P* = 0.014), but the incidence of bleeding events in the de-escalation group was significantly lower than that in the standard group (9.3% vs. 18.4%, *χ²*=7.191, *P* = 0.027). The Cox regression analysis showed that increases in haemoglobin (HGB) *(HR* = 0.986) and estimated glomerular filtration rate (eGFR) (*HR* = 0.983) could reduce the incidence of MACCEs, while old myocardial infarction (OMI) (*P* = 0.023) and hypertension (*P* = 0.013) were independent predictors of MACCEs.

**Conclusion:**

For STEMI patients undergoing PCI, the de-escalation scheme of ticagrelor to clopidogrel 75 mg or ticagrelor 60 mg at 3 months after PCI was related to the reduction of bleeding events, especially minor bleeding events, without an increase in ischaemic events.

## Introduction

Dual antiplatelet therapy (DAPT) refers to aspirin combined with a P2Y_12_ receptor inhibitor, and it is the cornerstone of the current preferred treatment to prevent ischaemic events in patients with acute coronary syndrome (ACS) [1]. The ISAR-REACT 5 study confirmed that prasugrel is preferred to ticagrelor in reducing ischaemic events in patients with ACS [2]. The current guidelines recommend that DAPT should be used for 12 months in patients with acute ST segment elevation myocardial infarction (STEMI) undergoing percutaneous coronary intervention (PCI), and that the P2Y_12_ receptor inhibitor clopidogrel should be preferred to the more powerful P2Y_12_ receptor inhibitors ticagrelor and prasugrel [3]. Although the antiplatelet pharmacodynamics and clinical efficacy of ticagrelor are better than those of clopidogrel [4] in patients with STEMI undergoing PCI, due to adverse drug reactions, an increased economic burden, an increased risk of bleeding and other factors, it is increasingly common for patients who are using ticagrelor to be forced to discontinue the drug and to reduce the level of treatment [5]. The TOPIC study confirmed that in patients with ACS undergoing PCI, a powerful P2Y_12_ inhibitor (ticagrelor or prasugrel) was changed to clopidogrel for 11 months after 1 month of therapy, which reduced the risk of bleeding without increasing the risk of ischaemia [6]. The PEGASUS-TIMI54 study confirmed that ticagrelor combined with low-dose aspirin (aspirin < 150 mg/d) can reduce the main adverse cardiac events (MACEs) in patients with stable coronary heart disease with an old myocardial infarction history and confirmed that the incidence of bleeding and dyspnoea in the 60 mg ticagrelor group was lower than that in the 90 mg ticagrelor group [7]. However, the above studies were carried out in European and American populations, and their applicability to Asian populations is unknown. In addition, the subjects were all patients with ACS or stable coronary heart disease. Evidence-based research on whether the de-escalation scheme is applicable to STEMI patients is still lacking. The purpose of this study was to investigate the effectiveness of the de-escalation treatment scheme of replacing ticagrelor with clopidogrel or reducing the dose of ticagrelor on the prognosis of patients with STEMI after PCI.

## Methods

### Study Population

**Fig. 1:**
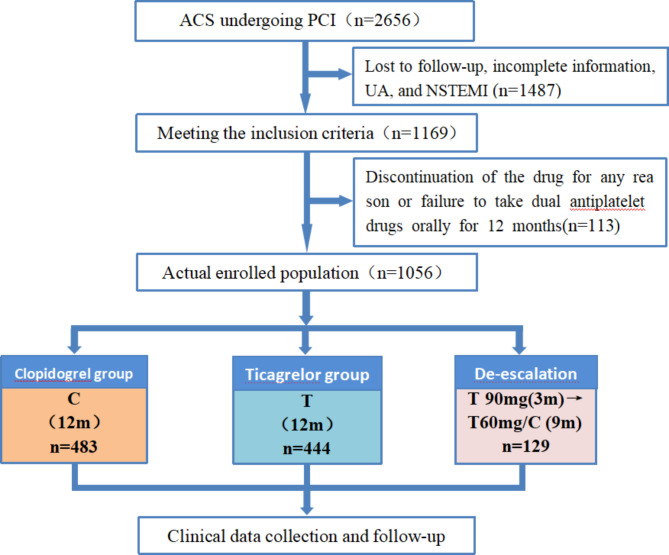
Study profile C?clopidogrel?T?ticagrelor?ACS?acute coronary syndrome?PCI?percutaneous coronary intervention

This study was a single-centre retrospective cohort study. The subjects were 1056 patients with STEMI who were hospitalized in the Department of Cardiology of Langfang People’s Hospital and received PCI treatment from March 2017 to August 2021, with 483 patients in the standard group, 444 patients in the intensive group, and 129 patients in the de-escalation group (Fig. 1). The diagnostic criteria were as follows: all STEMI patients included in this study met the relevant diagnostic criteria in the ESC guidelines [3]. The inclusion criteria were as follows: age ≥ 18 years and follow-up time more than 12 months. All patients had typical symptoms of STEMI, including ECG manifestations (it is typical practice to designate patients with persistent chest discomfort or other symptoms suggestive of ischaemia and ST-segment elevation in at least two contiguous leads as STEMI) or laboratory examination evidence (defined as an elevation of cardiac troponin values with at least one value above the 99th percentile upper reference limit), and had undergone PCI treatment. The exclusion criteria were as follows: patients allergic to aspirin or any P2Y_12_ receptor inhibitor or with serious adverse reactions (major bleeding, such as gastrointestinal bleeding or cerebral haemorrhage, significant bradycardia, and intolerable dyspnoea); patients who could not continue taking the medication, who discontinued the drug for any reason or who failed to take dual antiplatelet drugs orally for 12 months; patients who were complicated with diseases that seriously impacted platelet count and function, such as severe rheumatic immune diseases and aplastic anaemia; patients who had severe hepatic and renal insufficiency (Child‒Pugh grade 2 or 3 or eGFR < 30 ml/min/1.73 m^2^); and patients who participated in other research projects related to antiplatelet and anticoagulation during the follow-up period.

## Baseline data collection

A case report form containing the patient’s baseline data and prognosis follow-up results were designed for this study, and the baseline data of the patients were filled in by consulting their electronic medical records. The patient grouping and prognosis follow-up data were obtained and completed in the case report form through telephone follow-up and outpatient follow-up. After the completion of the case report form, the relevant data were separately entered into SPSS 21.0 data software (International Business Machines Corporation) by two people separately to ensure entry accuracy. The contents of the baseline data in this study were determined after consulting the literature and after discussion by the research team, and the discussion included the patients’ general information and treatment strategies during hospitalization, such as age, sex, body mass index (BMI), primary PCI or not, chronic medical history, laboratory tests, pathological changes in the culprit artery and procedure conditions. The primary end point was major adverse cardiovascular and cerebrovascular events (MACCEs) during follow-up, including cardiac death, myocardial infarction, ischaemia-driven revascularization, and stroke. The primary safety endpoint was bleeding events, including major bleeding and minor bleeding defined by thrombolysis in myocardial infarction (TIMI). The follow-up time was 12 months after PCI.

## Definitions

In this study, the patients in the de-escalation group took DAPT after PCI (aspirin 100 mg once daily + ticagrelor 90 mg twice daily), replaced ticagrelor with clopidogrel 75 mg once daily or ticagrelor 60 mg twice daily at 3 months after PCI, and continued to take DAPT for 9 months. The patients in the standard treatment group took DAPT after PCI (aspirin 100 mg once daily + clopidogrel 75 mg once daily) for 12 months. The patients in the intensive group took DAPT after PCI (aspirin 100 mg once daily + ticagrelor 90 mg twice daily) for 12 months without any reduction or change in medication.

### Bleeding events

The bleeding classification criteria used in this study were the TIMI bleeding classification criteria, including TIMI major bleeding and minor bleeding. TIMI major bleeding is defined as fatal haemorrhage (death due to haemorrhage), intracranial haemorrhage, or gastrointestinal haemorrhage requiring blood transfusion; minor bleeding is defined as epistaxis, gingival bleeding, bulbar conjunctival bleeding, skin ecchymosis, positive urine blood, faecal occult blood, etc., and does not require blood transfusion in clinical practice [8].

## Follow-up

All patients undergoing PCI were followed-up by a trained data clerk. The time of outpatient follow-up was the second week, the third month, the sixth month and the first year after PCI. The patients who did not receive outpatient follow-up were followed-up by telephone.

### Statistical analysis

SPSS 21.0 software was used for statistical analysis. The measurement data with a normal distribution are expressed as the mean ± standard deviation, and comparisons among the groups were analysed by one-factor ANOVA. The measurement data with a nonnormal distribution are expressed as the median and interquartile interval, and the comparisons among the groups were analysed by the rank sum test. The counting data were expressed as frequencies and percentages, and the chi-square test was used for analysis among the groups. Cox multivariate regression analysis was used to adjust the baseline data. Relevant covariates were selected as follows : variables with statistically significant differences by univariate analysis and relevant factors that may affect the outcome indicators of this study in clinical and previous studies. Kaplan‒Meier analysis was used to analyse the survival rate of the three groups of patients. Using bilateral tests, results in which *P ≤* 0.05 indicated that the difference among the three groups was statistically significant, and we used *P* < 0.017 to adjust for multiple comparisons of 3 pairwise comparisons.

## Results

### Baseline data

The analysis results showed that the age of the standard treatment group was higher than that of the intensive and de-escalation groups, and the differences were statistically significant (*P* < 0.001 and *P* = 0.001, respectively). However, the difference in age between the intensive and de-escalation groups was not statistically significant (*P* > 0.017). The proportion of female patients in the standard group was higher than that in the intensive and de-escalation groups, and the difference was statistically significant (*P* = 0.002 for each comparison). There was not a statistically significant sex difference between the intensive and de-escalation groups (*P* > 0.017). The proportion of patients with primary PCI in the intensive group was higher than that in the de-escalation group (*P* = 0.012). The proportion of patients with primary PCI in the standard group was not significantly different from that in the intensive group (*P* > 0.017). There were no statistically significant differences among the three groups in the other aspects of baseline data, including body mass index and chronic medical history (*P* > 0.05). The baseline data of the three groups are shown in Table 1.

**Table 1:** Baseline Characteristics

Characteristic	Standard Group (n = 483)	Intensive Group (n = 444)	De-escalation Group (n = 129)	*F*/*χ²* value	*P* value	
Age (years; m ± SD)	61.61 ± 11.16	57.90 ± 10.80	57.97 ± 11.88	14.530	0.000	(1)vs.(2), p<0.001; (1)vs.(3), p = 0.001;
Female (n, %)	149(30.8%)	95(21.4%)	22(17.1%)	16.130	0.000	(1)vs.(2), p = 0.002; (1)vs.(3), p = 0.002;
BMI (kg/m^2^; m ± SD)	25.68 ± 3.88	26.14 ± 4.42	26.39 ± 3.76	0.849	0.428	
Primary PCI (n, %)	380(78.7%)	374(84.2%)	97(75.2)	7,302	0.026	(1)vs.(2), p = 0.024; (2)vs.(3), p = 0.012;
Medical history (n, %)						
Hypertension	312(64.6%)	288(64.9%)	82(63.6%)	0.074	0.964	
Type 2 diabetes	107(22.2%)	103(23.2%)	24(18.6%)	1.223	0.543	
Cerebrovascular disease	64(13.3%)	47(10.6%)	12(9.3%)	2.382	0.304	
OMI	20(4.1%)	16(3.6%)	2(1.6%)	2.362	0.307	
Atrial fibrillation	14(2.9%)	12(2.7%)	4(3.1%)	0.068	0.967	
Current smoker	252(52.2%)	235(52.9%)	74(57.4%)	1.113	0.573	
CAD family history	8(1.7%)	7(1.6%)	6(4.7%)	4.127	0.127	
Peripheral artery disease	5(1.0%)	5(1.1%)	0(0.0%)	2.637	0.268	
Previous RV	23(4.8%)	31(7.0%)	11(8.5%)	3.405	0.182	

M, mean; SD, standard deviation; BMI, body mass index; PCI, percutaneous coronary intervention; OMI, old myocardial infarction; CAD, coronary artery disease; RV, revascularization

### Laboratory test results

The analysis results showed that there was no significant difference among the three groups for the laboratory test results, including platelet (PLT), fibrinogen (FIB), markers of myocardial injury, fasting blood glucose (FBG), blood lipids, N-terminal B type natriuretic peptide (NT-pro BNP) and other cardiac function indicators (*P* > 0.05).

The white blood cell (WBCs) counts of the standard group were lower than those of the intensive and de-escalation groups (*P* = 0.016 and *P* < 0.001, respectively). There was no statistically significant difference between the intensive and de-escalation groups. The haemoglobin (HGB) level of the standard group was lower than that of the intensive and de-escalation groups (*P* < 0.001). There was no statistically significant difference in HGB between the intensive and de-escalation groups (*P* > 0.017). The estimated glomerular filtration rate (eGFR) in the standard group was lower than that in the intensive group (*P* < 0.001), but there was no statistically significant difference between the other groups (*P* > 0.017). The uric acid (UA) levels in the standard group were lower than those in the intensive group (*P* = 0.002), but there was no statistically significant difference between the other groups (*P* > 0.017). The laboratory test results of the three groups are shown in Table 2.

**Table 2:** Laboratory Tests

Characteristic	Standard Group (n = 483)	Intensive Group (n = 444)	De-escalation Group (n = 129)	*F*/*χ²* value	*P* value	
WBC (×10^9^/L)	8.27 ± 2.83	8.79 ± 2.93	9.45 ± 3.89	7.584	0.001	(1)vs.(2), p = 0.016; (1)vs.(3), p<0.001; (2)vs.(3), p = 0.039;
HGB (g/L)	133.42 ± 19.66	139.46 ± 18.69	144.35 ± 19.78	18.451	0.000	(1)vs.(2), p<0.001 (1)vs.(3), p<0.001; (2)vs.(3), p = 0.017;
PLT (×10^9^/L)	237.88 ± 69.57	242.39 ± 69.97	245.33 ± 68.61	0.705	0.495	
FIB (g/L)	3.59 ± 1.02	3.43 ± 1.01	3.50 ± 1.04	2.063	0.128	
cTnI (ug/L)	3.60(0.16, 15.49)	5.84(0.64, 13.06)	2.40(0.45, 10.00)	2.152	0.341	
NT-pro BNP (ng/L)	836.00(300.50, 1611.50)	701.50(287.00, 1445.00)	366.00(88.50, 921.00)	2.440	0.295	
CK-MB (U/L)	47.98(12.52, 120.84)	60.00(12.68, 115.47)	46.00(16.75, 190.00)	2.212	0.331	
Cr (µmol/L)	68.41 ± 20.57403	66.32 ± 19.00	67.01 ± 15.91	1.101	0.333	
eGFR	95.50 ± 14.65	98.78 ± 15.56	98.85 ± 15.17	6.186	0.002	(1)vs.(2), p = 0.001; (1)vs.(3), p = 0.026;
UA (µmol/L)	321.77 ± 83.07	339.73 ± 88.78	331.15 ± 83.85	5.034	0.007	(1)vs.(2), p = 0.002;
FBG (mmol/L)	7.01 ± 2.49	7.169 ± 2.80	6.84 ± 2.44	0.625	0.536	
HbA1C (%)	6.76 ± 1.74	6.49 ± 1.54	6.54 ± 1.56	1.032	0.357	
TC (mmol/L)	5.00 ± 1.54	4.92 ± 1.37	4.91 ± 1.44	0.267	0.766	
TG (mmol/L)	1.85 ± 1.26	1.92 ± 1.18	1.60 ± 0.80	2.614	0.074	
LDL-C (mmol/L)	2.98 ± 0.99	2.87 ± 0.96	2.86 ± 0.88	0.486	0.615	
HDL-C (mmol/L)	1.03 ± 0.41	1.01 ± 0.47	1.03 ± 0.27	0.313	0.731	
Lp (a) (mg/L)	165.15 (89.125, 383.925)	163.75 (63.775, 397.925)	188.8 (143.9, 311.4)	2.2434	0.296	

HGB, haemoglobin; PLT platelet; FIB, fibrinogen; cTnI cardiac troponin I; NT-pro BNP, N terminal B type natriuretic peptide; eGFR, estimated glomerular filtration rate; FBG, fasting blood glucose; TC, total cholesterol; TG, triglyceride; LDL-C, low density lipoprotein-cholesterol; HDL-C, high density lipoprotein-cholesterol; Lp (a), lipoprotein (a)

### Coronary angiography and interventional procedure

The proportion of coronary ostial lesions in the de-escalation group was lower than that in the standard and intensive groups (*P* = 0.006 and *P* = 0.002, respectively), but there was no statistically significant difference between the standard andintensive groups (*P* > 0.017). The proportion of diffused lesions in the standard group was higher than that in the de-escalation group (*P* = 0.001), but there was no statistically significant difference between the standard and intensive groups (*P* = 0.017), and there was no statistically significant difference between the intensive and de-escalation groups (*P* > 0.017). The rate of in-stent restenosis (ISR) in the de-escalation group was higher than that in the standard group (*P* = 0.001), but there were no significant differences among the other groups (*P* > 0.017). The proportion of small vessel lesions in the de-escalation group was lower than that in the standard and intensive groups (*P* < 0.001), but there was no significant difference between the standard and intensive groups (*P* > 0.017). The rate of proximal segment of left anterior descending (LADp) in the de-escalation group was higher than that in the standard and de-escalation groups (*P* = 0.002 and *P* < 0.001, respectively), but there was no statistically significant difference between the standard and de-escalation groups (*P* > 0.017). There were no statistically significant differences among the three groups in terms of the other details of PCI, such as chronic total occlusion (CTO), the number of stents implanted, and complete revascularization (complete RV) (*P* > 0.05). The coronary angiography and interventional therapy results of the three groups are shown in Table 3.

**Table 3:** Coronary angiography and interventional therapy

Characteristic	Standard Group (n = 483)	Intensive Group (n = 444)	De-escalation Group (n = 129)	*F*/*χ²* value	*P* value	
Lesion artery (n, %)						
LM	19(4.0%)	13(3.0%)	5(3.9%)	0.782	0.676	
LAD	409(85.9%)	392(89.1%)	110(85.3%)	5.614	0.132	
LCX	328(68.9%)	280(63.6%)	78(60.5%)	4.569	0.102	
RCA	360(75.6%)	337(76.6%)	88(68.2%)	3.865	0.145	
Ostial lesion (n, %)	130(27.3%)	127(28.9%)	20(15.5%)	9.429	0.009	(1)vs.(3), p = 0.006; (2)vs.(3), p = 0.002;
Diffused lesion (n, %)	323(48.7%)	180(40.9%)	42(32.6%)	12.805	0.002	(1)vs.(2), p = 0.017; (1)vs.(3), p = 0.001;
CTO (n, %)	17(3.6%)	27(6.1%)	3(2.3%)	5.118	0.077	
ISR (n, %)	2(0.4%)	8(1.8%)	5(3.9%)	8.936	0.011	(1)vs.(2), p = 0.042; (1)vs.(3), p = 0.001;
Small vessel (n, %)	122(15.5%)	118(23.0%)	14(3.7%)	14.653	0.001	(1)vs.(3), p<0.001; (2)vs.(3), p<0.001;
LADp (n, %)	80(16.8%)	53(12.0%)	37(28.7%)	20.456	0.000	(1)vs.(2), p = 0.041; (1)vs.(3), p = 0.002; (2)vs.(3), p<0.001.
Number of stents (n; m ± SD)	1.37 ± 0.57	1.40 ± 0.64	1.37 ± 0.57	0.400	0.670	
Complete RV (n, %)	276(58.0%)	267(60.7%)	68(52.7%)	2.693	0.260	

PCI, percutaneous coronary intervention; LM, left main; LAD, left anterior descending; LCX, left circumflex coronary artery; RCA, right coronary artery; CTO, chronic total occlusion; ISR, in-stent restenosis; RV, revascularization; LADp proximal segment of left anterior descending

### Endpoints during follow-up

Compared with the standard and intensive groups, there were no statistically significant differences in the incidence of MACCEs between the patients in the de-escalation group during the follow-up period (*P* > 0.05), while the standard group had a significant increase in MACCEs compared with the intensive group (*P* = 0.014). The incidence of minor bleeding events in the de-escalation group was significantly lower than that in the intensive group (9.3% vs. 18.4%, *χ²* =7.191, *P* = 0.027). The endpoints during follow-up of the three groups are shown in Table 4.

**Table 4:** Endpoints during follow-up

Characteristic	Standard Group (n = 483)	Intensive Group (n = 444)	De-escalation Group (n = 129)	*F*/*χ²* value	*P* value	
MACCEs (n, %)	41(8.6%)	20(4.5%)	8(6.2%)	6.174	0.046	(1)vs.(2), p = 0.014;
MI (n, %)	12(2.5%)	10(2.3%)	3(2.3%)	0.063	0.969	
TVR (n, %)	16(3.4%)	12(2.7%)	2(1.6%)	1.250	0.535	
Stroke (n, %)	6(1.3%)	1(0.2%)	2(1.6%)	4.289	0.117	
Cardiac death (n, %)	19(4.0%)	9(2.0%)	3(2.3%)	3.218	0.200	
Bleeding events (n, %)	78(16.4%)	92(20.9%)	17(13.2%)	5.411	0.067	
Major bleeding	14(2.9%)	13(3.0%)	5(3.9%)	0.307	0.858	
Minor bleeding	68(14.3%)	81(18.4%)	12(9.3%)	7.191	0.027	(2)vs.(3), p = 0.014;

MACCEs, major adverse cardiovascular and cerebrovascular events; MI, myocardial infarction; TVR, target vessel revascularization

### COX regression results of prognosis of patients

The variables with different baseline and other relevant influencing factors are included in the COX regression equation as independent variables. The results showed that increases in HGB (*HR* = 0.986) and eGFR (*HR* = 0.983) could reduce the incidence of MACCEs, while OMI (*P* = 0.023) and hypertension (*P* = 0.013) were independent predictors of MACCEs. The risk of MACCEs in the patients with OMI and hypertension was 2.158 times (*HR* = 2.158) and 3.340 times (*HR* = 3.340) higher, respectively, than that in the patients without the above medical history. The risk of minor bleeding in the intensive group was 1.411 times higher than that in the other two groups (*HR* = 1.411). The COX regression analysis results of the other differential indicators, such as age, sex, primary PCI, WBC count, UA, ostial lesions, diffuse lesions, ISR lesions, small vessel lesions, and LADp, were not statistically significant. The Cox regression results for the prognosis of patients are shown in Table 5.

**Table 5:** Cox regression

Endpoints	Variables	Quotient		*Wald*	*HR*	95% CI	*P* value
		*B*	*SE*				
MACCEs	HGB (g/L)	-0.014	0.005	6.804	0.986	0.975–0.996	0.009
	eGFR	-0.017	0.007	5.972	0.983	0.970–0.997	0.015
	Hypertension	0.769	0.310	6.150	2.158	1.175–3.963	0.013
	OMI	1.206	0.405	8.865	3.340	1.510–7.389	0.003
Minor bleeding	Intensive Group	0.345	0.158	4.780	1.411	1.036–1.922	0.029

MACCE, major adverse cardiovascular and cerebrovascular events; OMI, old myocardial infarction; RV, revascularization

### Results of Kaplan‒Meier curve

**Fig. 2:**
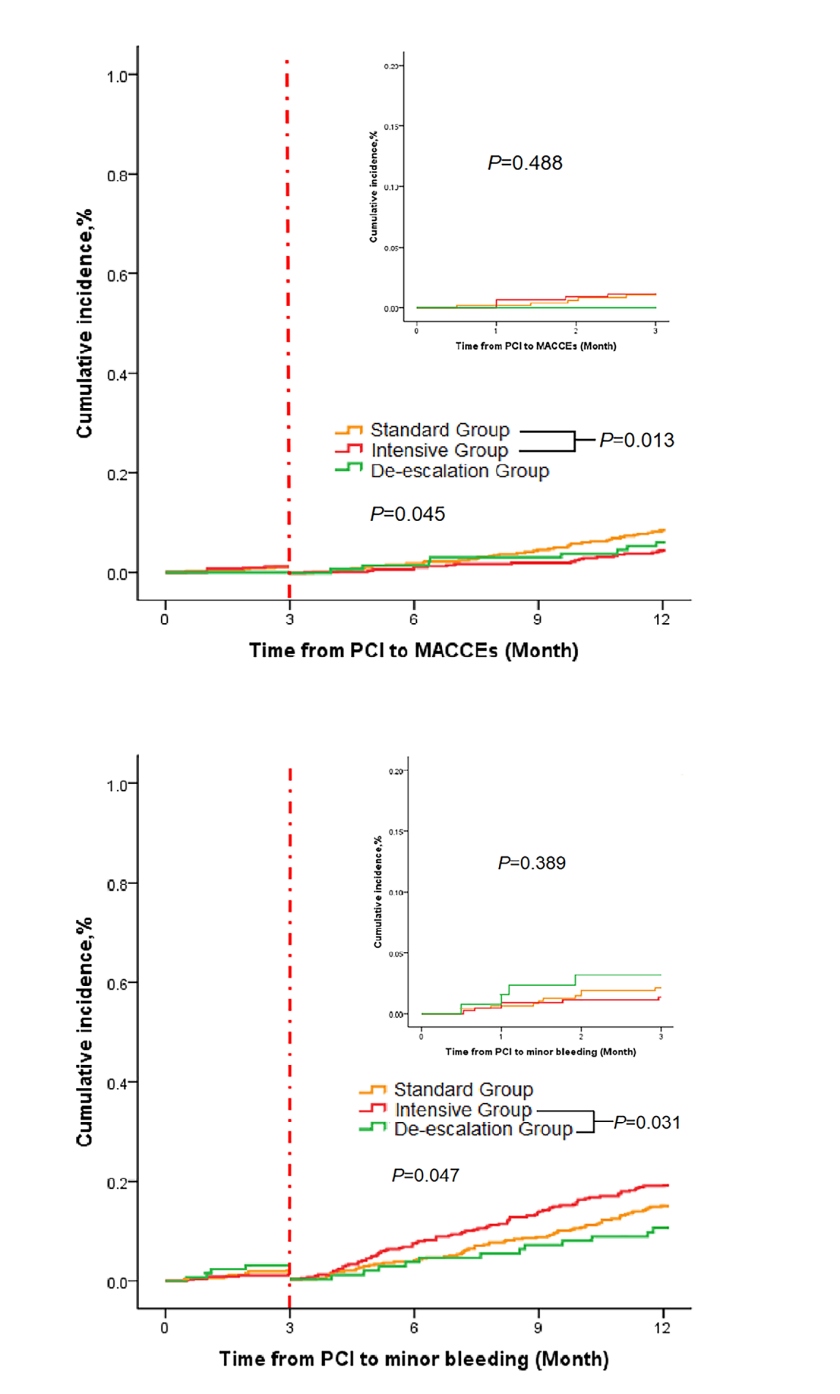
Kaplan?Meier estimates of the incidence of TIMI minor bleeding and MACCEs 12 months after PCI Figure 2(a) MACCE cumulative incidence; MACCE, major adverse cardiovascular and cerebrovascular events. MACCEs included the composite endpoints of cardiac death, myocardial infarction, ischaemia-driven revascularization, and stroke. Cut-off point: 3 months after percutaneous coronary intervention (PCI) Figure 2(b) Minor bleeding event cumulative incidence. The bleeding classification criteria used in this study were the TIMI bleeding classification criteria, including TIMI major bleeding and minor bleeding. TIMI minor bleeding is defined as epistaxis, gingival bleeding, bulbar conjunctival bleeding, skin ecchymosis, haematuria, or positive stool occult blood, none of which require treatment by blood transfusion. Cut-off point: 3 months after PCI

The Kaplan‒Meier survival curve showed that there was no significant difference in the incidence of MACCEs and TIMI minor bleeding among the three groups within 3 months after PCI, while the incidence of MACCEs in the standard group was significantly higher than that in the intensive group within 3–12 months after PCI (*P* = 0.013). The risk of TIMI minor bleeding in the de-escalation group was significantly lower than that in the intensive group within 3–12 months after PCI (*P* = 0.031). The results of the Kaplan‒Meier curve are shown in Fig. 2.

## Discussion

This was a real-world, single-centre cohort study. A total of 1056 patients with STEMI undergoing PCI were retrospectively analysed. The following conclusions were drawn: (1) The reduction in minor bleeding events within one year after PCI was related to the de-escalation treatment of 90 mg ticagrelor three months after PCI, while the incidence of MACCEs was not significantly increased. (2) The use of clopidogrel 75 mg in STEMI patients after PCI was related to the increasing incidence of MACCEs within one year compared with that of ticagrelor 90 mg. (3) Anaemia and renal insufficiency can increase the incidence of MACCEs, and OMI and hypertension are independent predictors of MACCEs.

The baseline data of this study showed that the age of the patients in the standard group was significantly higher than that in the intensive and de-escalation groups (*P* < 0.001 and *P* = 0.001, respectively), but there was not a statistically significant difference in the age of patients between the intensive and de-escalation groups (*P* > 0.017). The proportion of female patients in the standard group was higher than that in the intensive and de-escalation groups (*P* = 0.002 for both comparisons), but there was not a statistically significant sex difference between the intensive and de-escalation groups (*P* > 0.017). Because this study focused on the prognosis of antiplatelet drug de-escalation in the intensive and de-escalation groups, there were no significant differences in age and gender between the two groups; Although the age and proportion of female patients in the standard group were higher than those in the other two groups, the Cox regression analysis of age and sex did not show statistical significance, so age and sex differences were not considered as the key factors in the outcomes of this study.

At present, it is relatively common for ACS patients to receive de-escalation treatment with 90 mg ticagrelor. Approximately 5.3–13.6% of patients undergo early de-escalation in the hospital [9]. Several randomized controlled trials have explored the de-escalation treatment of ticagrelor. The PEGASUS-TIMI54 study was aimed at patients with stable coronary heart disease complicated with old myocardial infarction and confirmed the safety of a reduction in ticagrelor from 90 mg to 60 mg [7]. In the TWILLIGHT study, DAPT was reduced to a single drug treatment of ticagrelor at 3 months after PCI, and it was confirmed that the risk of bleeding could be reduced without increasing ischaemic events [10]. However, these studies did not include STEMI patients. The subjects of this retrospective cohort study were patients with STEMI because patients with STEMI have a higher risk of thrombosis load and ischaemic events. Our study also confirmed the safety of early de-escalation in this population.

This study confirmed that the de-escalation scheme of converting ticagrelor 90 mg to clopidogrel 75 mg or ticagrelor 60 mg 3 months after PCI significantly reduced the incidence of TIMI minor bleeding events. Although the PLATO study showed that the primary composite endpoint (including cardiovascular death, myocardial infarction and stroke) of the ticagrelor group was reduced by 16% compared with the clopidogrel group, it also showed a 10-fold increased the risk of intracranial haemorrhage (0.1% vs. 0.01%, *P* = 0.02) [11], and the incidence of bleeding was relatively higher in real-world patients who take ticagrelor. The TOPIC study confirmed that the de-escalation scheme of replacing powerful P2Y_12_ receptor inhibitors (ticagrelor or prasugrel) with clopidogrel one month after PCI in ACS patients reduced the incidence of bleeding events without increasing ischaemic events [6]. However, only 40% of the 646 ACS patients included were STEMI patients, the sample size was too small, and the definition of bleeding events using the BARC bleeding classification was relatively broad. If the TIMI bleeding classification was replaced, the bleeding results were no longer statistically significant. Post hoc analysis of the TALOS AMI study confirmed that ticagrelor increased the incidence of nuisance bleeding within 1 month after PCI compared with clopidogrel, thereby increasing the risk of BARC type 2, 3 or 5 bleeding at 6 months (*HR* = 1.94 [*95% CI*, 1.08–3.48]; *P* = 0.026) [12]. It can also be observed from the Kaplan‒Meier curve that the 1056 patients with STEMI involved in this study had a lower incidence of TIMI minor bleeding events after 3 months of de-escalation therapy, which compensates for the shortcomings of the aforementioned studies in terms of the broadness of bleeding evaluation criteria and small sample size and confirms the advantages of de-escalation therapy in terms of bleeding events.

According to the Kaplan‒Meier curve, the MACCEs in the standard group were significantly increased compared with those in the intensive group at 3–12 months after PCI, and this result was not completely consistent with the relevant results of the previous studies evaluating de-escalation from ticagrelor to clopidogrel. Although the COSTIC study confirmed that the incidence of net clinical adverse events of patients with ACS who received clopidogrel immediately after PCI was lower than that of patients who received ticagrelor within 12 months after discharge (5.4% vs. 8.3%, *HR* = 0.63, *95% CI*: 0.50 ~ 0.80) [13], STEMI patients accounted for only 22.3% of the subjects in this study, and the analysis showed that ticagrelor still had significant advantages in preventing ischaemic events at the early stage (within one month). However, the Kaplan‒Meier curve in this study showed that clopidogrel did not increase the incidence of early (3 months) MACCEs in patients with STEMI. This result may be related to the difference in the study population, risk level and subjective de-escalation. More than 50% of the patients with STEMI in the TALOS AMI study were converted to clopidogrel by a powerful P2Y_12_ receptor inhibitor one month later, which confirmed that there was no increase in ischaemic events (2.1% vs. 3.1%, *P* = 0.148) while reducing bleeding events (3.0% vs. 5.6%, *HR* = 0.52 *95% CI* 0.35–0.77 *P* = 0.001) [14]. However, the subjects selected in this study were patients without adverse events within one month after PCI, and patients with high risk were avoided subjectively, especially those with high ischaemic risk. By comparing the baseline data of the PCI patients, we found that the diffused lesions and ostial lesions in the de-escalation group were less than those in the intensive group, and these lesions can increase the incidence of ischaemic events, as confirmed by other research [15]. We also took into account the complexity of coronary artery disease and the risk of ischaemic events when guiding some patients to actively de-escalate. Powerful P2Y_12_ receptor inhibitors have been proven by many studies to reduce the incidence of long-term ischaemic events in ACS patients [10, 16], and it can also be observed through Kaplan‒Meier curves that the long-term use of clopidogrel increases the incidence of long-term MACCEs. Therefore, it is not advisable to cease individual risk assessment and conduct unified de-escalation treatment in STEMI patients undergoing PCI.

Because some patients have intolerable side effects, such as dyspnoea and bradyarrhythmia, when taking ticagrelor, they take clopidogrel for antiplatelet therapy after PCI. However, the Asian population is more likely to have multiple clopidogrel resistance [17], and the risk of ischaemia in STEMI patients is relatively high, which can increase the incidence of MACCEs. Therefore, in this real-world retrospective analysis of patients with STEMI, we concluded that clopidogrel could increase the incidence of MACCEs compared with ticagrelor. An observational study found that the risk of thrombosis was high in the early stage of ACS (within 30 days), and the risk of stent thrombosis continued to increase within 6 months after drug-eluting stent implantation, especially within 4 to 6 weeks after PCI [18, 19]. In addition, the median time for patients to be forced to stop ticagrelor was 44.5 ± 33.2 days, resulting in a relative increase in ischaemic risk during this period [5]. Therefore, we did not choose the timing of 1-month de-escalation that was commonly used in previous studies but instead adopted a 3-month de-escalation timing to avoid the period of high ischaemia risk and to simultaneously reduce the risk of minor bleeding.

The most common reasons among the nonbleeding side effects for discontinuation of ticagrelor were dyspnoea and bradyarrhythmia [20]. Passive de-escalation for patients in our study occurred due to the above side effects. In patients undergoing PCI, low compliance with P2Y_12_ receptor inhibitors was significantly associated with an increased risk of MACEs (*P* = 0.029). This correlation still existed after correcting for potential confounders. High compliance with P2Y_12_ receptor inhibitors significantly reduces the risk of MACEs (*HR* = 0.172, *P* = 0.021) [21, 22]. In our study, we chose a de-escalation scheme of converting ticagrelor 90 mg to clopidogrel 75 mg or ticagrelor 60 mg to effectively reduce side effects and increase compliance, which is one of the reasons why MACCE events did not increase significantly.

Studies have shown that ticagrelor 60 mg has a similar antiplatelet effect compared with 90 mg, and the incidence of bleeding and other side effects is lower [7]. However, there was no significant difference between the platelet function test results of the two doses of ticagrelor (*P* = 0.73) [23], but the blood concentration of ticagrelor could directly increase the risk of bleeding (322.6 ng/mL vs. 222.1 ng/mL, *P* < 0.001) [24]. The above conclusions also confirmed the efficacy and safety of ticagrelor 60 mg. Although there have been studies on the de-escalation of ACS patients, such as the OPTIMA study [25], which reduced ticagrelor from 90 mg to 60 mg, the sample size of that study wa only 65, and it mainly focused on the efficacy of platelet function monitoring, without evaluation of prognosis, which cannot be used as the basis for a de-escalation scheme. For the first time, we obtained the safety conclusion that the de-escalation scheme of converting ticagrelor 90 mg to ticagrelor 60 mg in STEMI patients can provide new options for the de-escalation treatment of STEMI patients.

We found that anaemia and renal insufficiency can increase the incidence of MACCEs, while old myocardial infarction and hypertension history were also found to be independent predictors of MACCEs through Cox regression analysis. This is consistent with the results of the current evaluation system for ischaemic events. For the GRACE score, OPT-CAD score and other scoring systems for predicting ischaemic events [26, 27], anaemia, renal insufficiency, hypertension or old myocardial infarction can all increase the risk score, thus affecting the survival rate of patients. For patients with anaemia and impaired renal function, we should pay attention to the treatment of anaemia and impaired renal function and formulate individualized treatment strategies while reasonably selecting antiplatelet drug de-escalation schemes. Although old myocardial infarction and hypertension were not statistically significant among the three groups in this study, the results of the Cox regression analysis suggested that for patients with high ischaemic risk factors, we should give them individualized de-escalation treatment after evaluation. Upgrading the intensity of antiplatelet drug treatment or prolonging the treatment time for patients with a high risk of ischaemia should be considered. Additionally, we should not only focus on reducing the risk of bleeding but also ignore the role of powerful P2Y_12_ receptor inhibitors in improving ischaemic events and long-term prognosis. In addition to the evaluation system for ischaemic events, the PRECISE-DAPT score as a bleeding risk assessment tool for patients receiving dual antiplatelet therapy is of great significance for clinical bleeding risk prediction, and studies have confirmed that the PRECISE-DAPT score with regard to in-hospital mortality was noninferior compared with the Thrombolysis in Myocardial Infarction risk score. The PRECISE-DAPT score may be a significant independent predictor of in-hospital mortality in patients with STEMI treated with pPCI [28]. In addition, a decrease in the left ventricular ejection fraction (≤ 40%) in the setting of ST-segment elevation myocardial infarction is a significant predictor of mortality in the young ST-segment elevation myocardial infarction population [29].

## Conclusion

For STEMI patients undergoing PCI, ticagrelor was reduced to clopidogrel or 60 mg ticagrelor at 3 months after PCI, which was related to a reduction in minor bleeding events without a concurrent increase in the incidence of ischaemic events.

## Limitations

There are some limitations in this study. First, this is a retrospective study with a small sample size, a short follow-up time, and a low incidence of primary endpoint events, which can all affect the overall test efficiency of this study. Second, the de-escalation scheme in this study was partly subjectively determined by doctors; the patients were not randomly divided; and the two groups of patients were not completely synchronized in terms of enrolment time. In addition, platelet function monitoring was not carried out for all patients with de-escalation treatment, and the monitoring results were used to guide the de-escalation plan. Finally, the risk assessment of ischaemia and bleeding was not sufficiently conducted for the subjects in this study, which may have an impact on the incidence of events. A subgroup analysis of hypertension and old myocardial infarction was not performed, and the specific type and time course of dual antiplatelet application were not proposed.

## Data Availability

All data generated or analysed during this study are included in this published article.
